# Time trend of Vigitel Brasil operation indicators (2006 to 2023)

**DOI:** 10.11606/s1518-8787.2025059006369

**Published:** 2025-06-11

**Authors:** Taciana Maia de Sousa, Luiza Eunice Sá da Silva, Laura Cordeiro Rodrigues, Thaís Cristina Marquezine Caldeira, Letícia de Oliveira Cardoso, Rafael Moreira Claro

**Affiliations:** I Universidade do Estado do Rio de Janeiro Instituto de Nutrição Departamento de Nutrição Social Rio de Janeiro RJ Brasil Universidade do Estado do Rio de Janeiro. Instituto de Nutrição. Departamento de Nutrição Social. Rio de Janeiro, RJ, Brasil; II Universidade Federal de Pelotas Faculdade de Medicina Pelotas RS Brasil Universidade Federal de Pelotas. Faculdade de Medicina. Pelotas, RS, Brasil; III Universidade Federal de Minas Gerais Faculdade de Medicina Departamento de Medicina Preventiva e Social Belo Horizonte MG Brasil Universidade Federal de Minas Gerais. Faculdade de Medicina. Departamento de Medicina Preventiva e Social. Belo Horizonte, MG, Brasil; IV Ministério da Saúde Secretaria de Vigilância em Saúde e Ambiente Departamento de Análises Epidemiológicas e Vigilância de Doenças e Agravos Não Transmissíveis Brasília DF Brasil Ministério da Saúde. Secretaria de Vigilância em Saúde e Ambiente. Departamento de Análises Epidemiológicas e Vigilância de Doenças e Agravos Não Transmissíveis. Brasília, DF, Brasil; V Universidade Federal de Minas Gerais Escola de Enfermagem Departamento de Nutrição Belo Horizonte MG Brasil Universidade Federal de Minas Gerais. Escola de Enfermagem. Departamento de Nutrição. Belo Horizonte, MG, Brasil

**Keywords:** Methodological study, Health Survey, Public Health Surveillance, Risk Factors, Brazil

## Abstract

**OBJECTIVE:**

To analyze the time trend of indicators from the Vigitel data collection operation between 2006 and 2023.

**METHODS:**

A methodological study that analyzed the temporal trend of three Vigitel operation indicators: eligibility rate, response rate and refusal rate, as well as the number of contacts answered according to days of the week and times of day. A series of linear regressions was used to identify temporal variations for the entire period (2006 to 2023) and for the final five years of this period (2018 to 2023).

**RESULTS:**

During the 17 editions analyzed, there was a decrease in the eligibility rate (-3.26 pp/year; p < 0.001) with the greatest magnitude in the most recent period (-12.23 pp/year; p = 0.010). Although the inclusion of mobile phones in the register in 2023 has mitigated the drop in eligibility, especially in recent years, the reduction has remained high (-10.43 pp/year; p = 0.020). The response rate also fell over the entire period (-0.78; p < 0.001) and, together with the fall in eligibility, represents an obstacle to the sustainability of the survey. On the other hand, the refusal rate also fell (-0.23 pp/year; p = 0.030), indicating the consistent quality of the system’s operation. The number of contacts answered was concentrated on weekdays and between 9am and 8pm.

**CONCLUSIONS:**

An analysis of the data from the Vigitel survey suggests that the methodology used has become fatigued, with reductions in eligibility and response rates over the years. Only the refusal rate, despite being far from its lowest levels, remains at satisfactory levels. The search for alternatives that make participation more convenient for the respondent is an important path for surveys of this nature, with a view to maintaining the quality of health surveillance in the country.

## INTRODUCTION

Improving global health systems requires continuous evaluation of solid, quality data, obtained through information systems and health surveys that are updated to accurately reflect the health conditions of the population^1^. In recent decades, through the efforts of international organizations, governments and research institutions, the use of health surveys in low- and middle-income countries has been expanded^1^. In Brazil, especially since the 1980s, primary data has been collected through population surveys, which are recognized as fundamental components of a national health information system^3^.

Among Brazilian health surveys, the Surveillance System for Risk and Protective Factors for Chronic Diseases by Telephone Survey (Vigitel) stands out for having been carried out since 2006, covering a representative sample of the adult population (≥ 18 years) living in all the capitals of the 26 Brazilian states and the Federal District^6^. The conception of Vigitel in the early 2000s was driven by the epidemiological panorama of the time, when analyses of historical mortality series in the capitals of the Brazilian states indicated a more than threefold increase in the proportion of deaths from chronic non-communicable diseases (CNCDs) between 1930 and 2006^7^.

Since then, in 17 editions, Vigitel has been the survey responsible for continuously monitoring the temporal trend in the prevalence of CNCDs and their main risk factors in Brazilian capitals. The data collected annually remains a solid indicator of the evolution of health indicators, essential for monitoring actions to prevent and control CNCDs, such as the Strategic Action Plan for Tackling Chronic Non-Communicable Diseases in Brazil, 2011 to 2022^8^, and its most current version, 2021 and 2030^9^.

To provide an accurate picture of the health situation in Brazil, it is crucial that Vigitel be methodologically revised based on the observation of a possible drop in the quality of the information generated. It is worth noting that, since its conception, Vigitel’s sampling and data collection methodology has remained very similar to that adopted in its first edition, indicating its robustness over time. From 2006 to 2021, interviews were conducted exclusively by telephone^10^. However, the progressive reduction in landline telephone coverage in the country, especially in the capitals of the North and Northeast regions, pointed to the imminent need to incorporate a new data collection method into Vigitel^11^.

Data from household surveys in Brazil already pointed to this deterioration in landline coverage. In 2019, only 30.4% of households in Brazilian state capitals had a landline telephone, a significant reduction compared to 2013 (48.9%)^12^. These results also showed a disparity in coverage between the capitals, with only 9.2% in Macapá and 53.2% in Rio de Janeiro^12^. A previous study reported a decrease in the eligibility rate of landlines in the country^13^, making it unfeasible to carry out the survey exclusively using this mode. In response to this situation, in 2023, the Ministry of Health (MS) incorporated mobile phone interviews into the survey. However, to date, no studies have been carried out to provide a comprehensive analysis of Vigitel’s operation.

The aim of this study was therefore to analyze the time trend of the main indicators of the Vigitel data collection operation between 2006 and 2023.

## METHODS

This is a methodological study based on information recorded during the operation of Vigitel between 2006 and 2023. All the information analyzed was provided by the company contracted to collect the data and sent weekly to the MS to monitor the survey’s progress over the years.

### Background to Vigitel’s Sampling and Data Collection

Since 2006, Vigitel has interviewed approximately 54,000 individuals aged 18 or over every year, distributed in around 2,000 interviews in each of the capitals of the 26 Brazilian states and the Federal District. However, as of 2020, due to the COVID-19 pandemic, an emergency measure was implemented, reducing the sample to 1^,^000 individuals in each location^10,14^. In 2023, a further reduction was necessary due to administrative and operational issues, with a sample of around 800 individuals per capital^6^.

The survey sampling process is carried out in two stages. In the first, a predetermined number of 10,000 telephone lines are drawn in each city. Next, the selected lines in each city are resampled into replicas of 200 lines. With this, each replica should represent a similar proportion of lines by city region or telephone prefix to the total sample. Results of pilot studies carried out previously with mobile phone listings obtained by RDD (random digit dialing) indicated the low quality of mobile phone registrations. We therefore opted for replicates of 500 lines for this modal.

The second stage begins by identifying the lines drawn that are eligible for the system, i.e. active residential lines (methodology applied between 2006 and 2021). Lines assigned to companies, not in use or out of service were not considered eligible for the system. In addition, lines that do not answer six calls (between 2006 and 2008 up to 10 calls were made), which are made on different days and times, including Saturdays and Sundays and at night, and which probably correspond to unoccupied households, were also considered ineligible.

For each eligible landline, once the respondent has agreed to take part in the survey, the adult residents of the household are enumerated and then a random draw is made to select one of these adults for the interview. If the person drawn is present and available, the interview is carried out at the initial contact. Otherwise, the interview could be scheduled for a more convenient day and time. Refusal to take part in the study at each of these two moments is recorded and added up to make up the indicator of refusal to take part in the study.

A similar methodology was implemented for the operation with mobile phones (adopted from 2023), without the need for an intra-household lottery after confirming eligibility. This procedure is necessary in the case of landlines, since the line is owned by the household, but it is not necessary in the case of mobile phones, since ownership tends to be individual, and use is not shared between family members^15^.

### Quality Indicators for the Register and System Performance

Throughout its editions, Vigitel has monitored the following system quality indicators: 1) Eligibility rate: percentage of lines considered eligible for Vigitel among the complete set of lines drawn; 2) Response rate: percentage of interviews actually carried out among the drawn lines considered eligible; and 3) Refusal rate: percentage of eligible lines that refused to participate in the study, including both refusals to interview and refusals to schedule.

Since different methodologies for calculating these rates in telephone surveys are available, Vigitel has opted to use the standard American Association for Public Research (AAPOR) #2 methodology since 2006. According to this methodology, the success and refusal rates are estimated considering only the lines identified as eligible, and lines with undefined status are assumed to be ineligible^16^.

In addition, information on the most effective days and times in the 2023 operation is included. A total of 60 interviewers took part in the operation, 40 from 8.45am to 3.15pm and 20 from 3.15pm to 9.30pm. At weekends, a single shift of interviewers worked from 9:20 to 15:40.

### Data Organization and Analysis

All the data were organized and analyzed using Stata software^®^ version 16.0^17^.

Initially, data relating to the system’s three quality indicators, as well as the number of contacts answered according to days of the week and times of day, presented in the previous section, were estimated based on the operation’s microdata provided by the company responsible for collecting the data. This operation was carried out for each of the cities, for all the cities in the same geographical region (North, Northeast, Central-West, South, and Southeast) and for the entire set of cities studied by Vigitel.

Subsequently, a series of linear regressions were used to identify significant variations over the period studied. In each regression model, the Vigitel quality indicator in question was considered the outcome variable, while the year the survey was carried out was treated as the exposure variable. In this type of analysis, the angular coefficient of the regression model (Beta value) indicates the average variation of the indicator in percentage points per year (pp/year) and the P value identifies whether this variation was statistically significant (p < 0.05). This analysis of variation was carried out for the entire Vigitel period (from 2006 to 2023) and for the final five years of this period (from 2018 to 2023). In addition, an analysis of variation was carried out for a section of the complete period, excluding the years 2006, 2007, and 2008 (supplementary material^a^), due to the changes that occurred in the operation of the survey in its initial years, which could bias the comparison in the complete set of the period. It should be noted that there is no evidence of error in the data for this period, however, until 2009 the rates were calculated manually, and after that were calculated automatically.

^a^Supplementary material available from: https://www.scidb.cn/en/detail?dataSetId=2282546d1f0e43feb9b79fa6bfc6cd8c

Given the inclusion of mobile phones in the survey in 2023, three additional analyses were carried out. The first considered only the landline telephones in Vigitel 2023, the second only the mobile telephones and the third combined landline and mobile telephones in a summary table.

Finally, the data on the days and times when the most interviews took place was organized in the form of tables containing heat maps. Once again, the presentations were divided between landline and mobile phone charts. These figures were tabulated in raw form, without considering the variation in the number of interviewers on the different days and times, as the number and distribution of interviewers was not constant during the operation. The Vigitel 2023 operation began with six interviewers, gradually increasing to 60 interviewers during the first 45 days of collection. The initial expectation was to have shifts of a similar size in terms of the number of interviewers. However, due to the low conversion of landline interviews, the possibility of reducing the number of workers on the second shift was indicated, making it possible to reduce the costs of the operation. Therefore, in the respective section, number and distribution of interviewers for the period February to April 2023 is also presented.

## RESULTS

### Eligibility rate

During the entire period studied, the eligibility rate of landlines drawn for inclusion in Vigitel varied between 72.5% and 5.3%. When considering only the 2023 operation, the mobile phone register had an eligibility rate almost three times higher than that of the landline register (21.2% versus 5.3%). The composition of an estimate involving both registries resulted in an eligibility rate of 11.3%, mitigating the sharp drop seen in recent years (Table 1).

**Table 1:** Percentage (%) of eligible telephone lines in Brazilian state capitals and the Federal District identified from the total number of lines drawn for system operation, by year. Vigitel Brasil, 2006 to 2023.

Location	Year											Average change over the period (pp/year)					
	2006	2009	2012	2015	2018	2019	2020	2021	2023^c^	2023^d^	2023^e^	2006/23^c^	2018-2023^c^	2006-2023^d^	2018-2023^d^	2006-2023^e^	2018-2023^e^
Northeast	81.3	65.5	63.1	69.7	45.0	44.0	35.0	14.6	6.7	20.8	12.8	**-3.58**	**-13.22**	**-3.30**	-9.01	**-3.46**	**-11.40**
Aracaju	88.9	67.3	61.9	70.8	45.5	45.2	36.2	16.2	6.6	25.2	14.0	**-3.76**	**-13.60**	**-3.40**	-8.02	**-3.62**	**-11.37**
Fortaleza	84.7	65.8	68.8	71.4	50.8	46.1	41.0	19.8	7.7	17.2	12.6	**-3.43**	**-13.65**	**-3.24**	-10.78	**-3.33**	**-12.16**
João Pessoa	78.8	67.4	62.3	74.8	35.6	38.8	24.4	7.2	4.8	21.4	11.0	**-4.07**	**-11.92**	**-3.74**	-6.94	**-3.95**	-10.06
Maceio	77.4	63.5	60.2	74.0	44.3	42.9	32.9	9.9	7.6	16.8	11.9	**-3.56**	**-12.89**	**-3.38**	-10.15	**-3.48**	-11.61
Christmas	80.7	65.5	65.8	64.8	35.6	43.4	34.1	12.4	7.0	17.3	12.1	**-3.70**	**-13.08**	**-3.49**	-9.99	**-3.60**	-11.56
Recife	80.9	65.7	64.0	65.5	49.1	47.9	42.3	17.7	5.9	26.7	13.5	**-3.39**	**-15.06**	**-2.98**	-8.83	**-3.24**	**-12.79**
Salvador	81.5	63.2	69.8	74.5	57.3	50.9	45.1	18.8	8.7	18.8	13.9	**-3.10**	**-15.31**	**-2.91**	-12.27	**-3.00**	**-13.75**
São Luís	76.5	64.1	54.6	60.4	40.4	38.9	27.2	12.6	6.5	21.6	13.2	**-3.64**	**-11.17**	**-3.35**	-6.63	**-3.51**	-9.16
Teresina	82.3	67.2	60.4	71.4	46.2	41.7	32.1	17.1	5.7	21.9	13.1	**-3.54**	**-12.31**	**-3.23**	-7.44	**-3.40**	**-10.10**
North	68.6	59.4	54.7	61.2	33.8	26.8	18.8	9.9	3.7	21.6	8.7	**-3.52**	**-7.84**	**-3.17**	-2.45	**-3.42**	**-6.32**
Bethlehem	84.6	66.0	55.8	55.4	43.2	36.3	35.2	13.6	5.4	26.0	13.3	**-3.71**	-11.42	**-3.30**	-5.25	**-3.55**	-9.07
Boa Vista	78.7	60.4	55.2	60.1	33.7	20.1	13.0	9.0	2.2	22.8	6.3	**-4.32**	**-5.78**	**-3.92**	0.39	**-4.24**	**-4.56**
Macapá	68.7	62.2	53.5	59.6	31.4	21.8	10.2	8.7	2.4	19.0	6.2	**-4.03**	-5.98	**-3.70**	-1.01	**-3.95**	-4.83
Manaus	78.3	61.8	49.6	57.2	30.1	26.1	19.4	6.9	3.4	20.8	8.0	**-4.10**	**-8.07**	**-3.76**	-2.84	**-4.01**	-6.70
Palmas	52.3	54.6	60.9	62.7	26.6	23.3	12.7	8.3	3.8	20.9	8.7	**-2.81**	**-6.27**	**-2.47**	-1.15	**-2.71**	-4.81
Porto Velho	58.1	55.3	58.4	67.7	36.5	29.9	21.3	11.7	3.7	21.9	8.7	**-2.81**	**-8.84**	**-2.45**	-3.37	**-2.71**	**-7.31**
Rio Branco	59.8	55.5	49.8	65.4	35.2	30.1	20.2	11.0	4.9	20.1	10.0	**-2.86**	**-8.50**	**-2.56**	-3.92	**-2.76**	**-6.94**
Southeast^a^	77.8	50.1	66.8	55.7	55.5	49.5	44.2	22.8	7.8	23.1	14.4	**-2.86**	**-14.64**	**-2.55**	-10.04	**-2.73**	**-12.65**
Belo Horizonte	87.7	71.0	69.7	73.7	62.1	53.3	46.7	26.8	8.8	19.8	14.2	**-3.16**	**-15.33**	**-2.94**	**-12.04**	**-3.05**	**-13.73**
Rio de Janeiro	77.4	63.8	67.3	72.9	58.9	50.4	43.2	19.3	6.7	26.4	14.6	**-3.00**	**-15.49**	**-2.61**	-9.59	**-2.84**	**-13.13**
São Paulo	72.1	57.2	63.5	67.4	56.5	46.0	43.7	22.6	8.5	25.7	16.8	-2.10	**-13.38**	-1.77	-8.21	-1.94	-10.88
Victoria	74.2	65.0	66.7	75.4	44.5	48.1	43.0	22.4	7.1	20.7	12.1	**-3.16**	**-14.36**	**-2.90**	-10.31	**-3.06**	**-12.86**
Central-West^a^	60.8	53.1	60.9	64.9	45.6	43.6	36.0	18.9	6.6	19.5	11.9	**-1.95**	**-12.81**	**-1.70**	-8.96	**-1.85**	**-11.22**
Campo Grande	64.1	50.8	56.3	61.2	39.0	41.4	30.7	17.6	4.6	17.7	9.6	**-2.08**	**-12.37**	**-1.82**	-8.42	**-1.98**	**-10.87**
Cuiabá	56.4	53.9	56.3	65.4	32.9	37.7	28.0	16.0	4.6	22.2	10.3	**-2.21**	**-11.13**	**-1.87**	-5.85	**-2.10**	**-9.41**
Federal District	62.9	50.4	65.1	68.5	57.5	45.7	41.5	18.8	8.8	18.8	13.8	-1.74	**-13.35**	-1.54	-10.35	-1.64	**-11.86**
Goiânia	59.8	57.1	66.1	64.5	53.1	49.7	43.7	23.2	8.6	19.1	14.0	**-1.76**	**-14.37**	**-1.55**	**-11.21**	**-1.65**	**-12.74**
South	63.3	54.1	63.4	64.4	49.4	45.3	39.4	20.6	7.5	23.1	14.6	**-1.95**	**-13.22**	**-1.65**	-8.55	**-1.81**	**-11.08**
Curitiba	64.9	57.9	65.2	66.9	55.2	49.7	44.9	24.0	7.8	22.6	15.0	**-1.85**	**-14.69**	**-1.56**	-10.23	**-1.71**	**-12.53**
Florianópolis	63.5	53.2	64.2	63.8	41.2	42.2	36.5	19.1	7.5	21.8	13.9	**-2.01**	**-12.15**	**-1.74**	-7.87	**-1.89**	**-10.23**
Porto Alegre	61.6	51.1	60.8	62.4	52.0	43.9	36.8	18.6	7.2	24.8	15.0	**-1.99**	**-12.82**	**-1.64**	-7.55	**-1.83**	**-10.48**
Total^b^	72.5	58.5	61.2	64.1	44.2	40.4	32.8	15.9	5.3	21.2	11.3	**-3.26**	**-12.23**	**-2.95**	-7.45	**-3.14**	**-10.43**

^a^ Average values for the capitals of each region.

^b^ Average values for the 26 capitals and the Federal District.

^c^ Data only for the landline register in 2023.

^d^ Data only for the mobile phone register in 2023.

^e^ Combined data from the landline and mobile phone register in 2023.

Statistically significant values are shown in bold (p < 0.05).

Considering only the landline registers for the 2023 operation, there was an average reduction in the eligibility rate of 3.26 pp/year (p < 0.001) in the period between 2006 and 2023. However, the reduction observed in the most recent period (2018 to 2023) was more than three times higher (12.23 pp/year; p = 0.010). When both registrations are considered, the reduction in the most recent period was 10.43 pp/year (p = 0.020). Although lower than in the case of landlines, this is still a significantly high figure (Table 1).

Considering landline registers for the whole period, reductions were seen mainly in the capitals of the Northeast (-3.58 pp/year; p < 0.001) and North (-3.52 pp/year; p < 0.001) regions of the country. In the most recent period, a greater magnitude of reduction was observed in the capitals of the Southeast region (-14.64 pp/year; p = 0.010) (Table 1).

### Response Rate

Over the whole period, the response rate varied from 71.5% in 2006 to 58.7% in 2023, when only landline registers were considered (-0.78pp/year; p < 0.001). For the year 2023, the mobile phone registry had a response rate around 60% lower than the landline registry (24.1% versus 58.7%) (Table 2).

**Table 2:** Percentage (%) of response among eligible telephone lines drawn in Brazilian state capitals and the Federal District, by year. Vigitel Brasil, 2006 to 2023.

Location	Year											Average change over the period (pp)					
	2006	2009	2012	2015	2018	2019	2020	2021	2023^c^	2023^d^	2023^e^	2006-2023^c^	2018-2023^c^	2006-2023^d^	2018-2023^d^	2006-2023^e^	2018-2023^e^
Northeast	71.2	74.8	64.4	70.7	70.7	70.7	57.6	60.7	57.9	23.3	33.1	**-0.71**	-3.54	**-1.39**	-13.94	**-1.20**	-11.00
Aracaju	67.3	74.9	64.5	70.8	71.0	73.2	58.2	59.5	56.8	22.2	32.0	-0.60	-4.80	**-1.27**	-15.16	**-1.08**	-12.24
Fortaleza	70.1	72.8	64.5	70.1	70.4	74.2	58.3	60.6	60.7	25.3	35.7	**-0.57**	-3.82	**-1.26**	-14.45	**-1.06**	-11.32
João Pessoa	71.0	74.5	66.9	70.3	72.1	68.2	58.6	70.7	57.9	21.8	31.6	**-0.57**	-1.90	**-1.28**	-12.73	**-1.08**	-9.78
Maceio	72.5	75.4	65.8	71.3	70.2	68.7	54.3	56.2	54.1	28.1	37.0	**-0.92**	-4.20	**-1.43**	-11.99	**-1.26**	-9.33
Christmas	73.4	77.3	64.5	70.7	70.1	68.4	58.9	63.4	57.5	23.9	33.8	**-0.80**	-2.81	**-1.46**	-12.89	**-1.27**	-9.92
Recife	73.2	72.8	65.3	69.7	70.1	73.9	59.3	61.4	59.1	23.0	33.0	**-0.65**	-4.22	**-1.36**	-15.06	**-1.16**	-12.04
Salvador	68.5	76.1	63.5	70.8	70.9	74.8	53.5	55.5	51.3	22.4	31.2	**-0.96**	-6.83	**-1.53**	-15.51	**-1.35**	-12.87
São Luís	73.0	74.7	61.3	72.2	70.9	68.1	54.7	57.5	57.9	22.0	31.8	**-0.92**	-2.79	**-1.62**	-13.57	**-1.43**	-10.62
Teresina	71.8	74.8	62.8	70.1	70.4	67.2	62.9	61.2	66.2	20.8	31.5	-0.42	-0.48	**-1.31**	-14.11	**-1.10**	-10.89
North	74.3	77.1	65.3	70.8	71.0	64.8	53.4	63.0	56.3	23.9	33.5	**-1.05**	-1.57	**-1.68**	-11.28	**-1.49**	-8.41
Bethlehem	70.3	76.2	60.8	72.3	72.3	66.0	53.1	56.4	53.1	17.6	26.6	**-0.99**	-3.54	**-1.68**	-14.18	**-1.51**	-11.50
Boa Vista	71.3	75.7	61.4	70.3	74.1	64.7	51.1	61.3	57.7	22.8	32.7	**-0.85**	-1.09	**-1.53**	-11.56	**-1.34**	-8.59
Macapá	73.3	73.5	61.6	70.3	66.4	58.7	54.1	56.8	53.5	22.5	31.6	-1.45	-1.29	**-2.06**	-10.58	**-1.88**	-7.86
Manaus	71.5	74.3	65.7	70.1	72.0	66.7	50.9	70.0	55.0	25.3	34.6	**-0.77**	-1.62	**-1.35**	-10.50	**-1.16**	-7.71
Palmas	81.1	87.7	73.1	72.6	70.3	70.1	56.1	68.8	57.8	26.7	36.5	**-1.30**	-2.42	**-1.92**	-11.77	**-1.72**	-8.81
Porto Velho	78.0	76.2	68.0	70.4	70.2	63.1	55.3	60.9	59.9	25.5	35.9	**-1.15**	-0.38	**-1.82**	-10.71	**-1.62**	-7.59
Rio Branco	74.3	76.0	66.7	69.9	71.5	64.1	53.2	67.0	57.3	27.1	36.8	**-0.81**	-0.64	**-1.40**	-9.70	**-1.21**	-6.80
Southeast^a^	68.4	76.4	64.1	70.3	71.4	70.7	61.0	59.8	59.0	25.8	35.8	**-0.64**	-3.63	**-1.29**	-13.59	**-1.10**	-10.60
Belo Horizonte	71.7	83.4	68.4	71.6	72.5	71.8	67.2	62.3	61.7	28.7	39.2	**-0.75**	**-3.52**	**-1.40**	-13.42	**-1.19**	-10.27
Rio de Janeiro	65.0	75.1	62.8	69.0	70.0	68.1	52.9	55.4	54.0	20.5	29.8	**-0.80**	-3.99	**-1.45**	-14.02	**-1.27**	-11.25
São Paulo	65.5	73.2	62.2	70.8	72.6	71.9	64.5	62.2	63.8	22.6	33.4	-0.28	-2.66	-1.08	-15.03	-0.87	-11.79
Victoria	71.4	74.0	62.9	69.8	70.3	71.0	59.3	59.4	56.5	31.4	40.7	**-0.74**	-4.33	**-1.23**	-11.88	**-1.05**	-9.08
Central-West^a^	72.6	78.5	67.8	70.3	72.5	70.6	61.7	61.0	62.8	27.4	38.1	**-0.76**	-2.43	**-1.45**	-13.03	**-1.24**	-9.83
Campo Grande	68.9	79.3	70.5	71.4	71.2	68.0	61.0	62.0	63.4	26.9	37.7	**-0.67**	-1.29	**-1.38**	-12.25	**-1.17**	-9.01
Cuiabá	73.1	77.9	67.3	69.7	74.6	68.5	59.9	61.4	63.2	27.1	38.0	**-0.71**	-1.42	**-1.41**	-12.25	**-1.20**	-9.00
Federal District	68.4	76.6	65.3	69.8	72.7	74.9	57.8	59.3	61.4	29.1	39.5	**-0.81**	-3.90	**-1.45**	-13.57	**-1.24**	-10.48
Goiânia	79.8	80.0	68.1	70.6	71.4	71.2	68.1	61.5	63.0	26.5	37.3	**-0.84**	-3.12	**-1.56**	-14.07	**-1.34**	-10.83
South	68.4	78.8	63.8	70.9	71.1	69.6	60.4	60.5	63.3	24.0	34.8	**-0.56**	-1.87	**-1.33**	-13.68	**-1.12**	-10.43
Curitiba	71.3	79.1	67.3	71.2	71.7	71.3	66.0	61.9	67.8	24.3	35.9	**-0.62**	-1.48	**-1.47**	-14.53	**-1.25**	-11.03
Florianópolis	69.4	78.7	61.9	71.5	71.6	68.5	57.6	59.5	60.7	25.1	35.5	**-0.68**	-2.14	**-1.37**	-12.82	**-1.17**	-9.71
Porto Alegre	64.5	78.7	62.2	70.1	70.1	69.0	57.5	60.2	61.4	22.5	32.9	-0.38	-1.98	-1.15	-13.67	-0.94	-10.54
Total^b^	71.5	76.6	65.0	70.6	71.2	69.0	57.9	61.2	58.7	24.1	34.2	**-0.78**	-2.78	**-1.46**	-13.15	**-1.26**	-10.12

^a^ Average values for the capitals of each region.

^b^ Average values for the 26 capitals and the Federal District.

^c^ Data only for the landline register in 2023.

^d^ Data only for the mobile phone register in 2023.

^e^ Combined data from the landline and mobile phone register in 2023.

Statistically significant values are shown in bold (p < 0.05).

Regarding landline registers, the greatest reduction in the response rate was observed in the capitals of the northern region of the country (-1.05 pp/year; p < 0.001), with a stable trend in all regions in the most recent period (Table 2).

### Refusal rate

Over the entire period analyzed, the refusal rate evolved favorably, with values ranging from 9.1% in 2006 to 5.3% in 2023, when considering only landlines. Similar refusal rates were also observed for mobile phones (4.1%). In both scenarios, the average reduction in the refusal rate over the period was around 0.23 pp/year (p = 0.030) (Table 3).

**Table 3:** Percentage (%) of refusals* among eligible telephone lines drawn in Brazilian state capitals and the Federal District, by year. Vigitel Brasil, 2006 to 2023.

Location	Year											Average change over the period (pp)					
	2006	2009	2012	2015	2018	2019	2020	2021	2023^c^	2023^d^	2023^e^	2006-2023^c^	2018-2023^c^	2006-2023^d^	2018-2023^d^	2006-2023^e^	2018-2023^e^
Northeast	8.8	3.1	9.1	2.3	4.1	1.7	0.4	2.4	5.3	4.1	4.4	**-0.22**	1.27	**-0.25**	0.90	**-0.24**	0.99
Aracaju	11.3	4.1	9.1	2.9	4.2	1.2	0.1	2.0	6.5	5.0	5.4	**-0.29**	1.78	**-0.32**	1.33	**-0.31**	1.45
Fortaleza	10.1	3.6	8.7	2.1	3.7	1.3	0.6	2.6	4.9	3.7	4.1	**-0.30**	1.25	**-0.32**	0.91	**-0.32**	1.01
João Pessoa	10.3	3.3	11.9	3.2	4.7	2.5	0.5	2.2	6.1	3.8	4.4	-0.24	1.24	**-0.28**	0.55	-0.27	0.74
Maceio	7.2	3.0	9.9	2.0	4.6	1.7	0.5	2.6	3.8	4.6	4.3	-0.22	0.84	-0.21	1.07	-0.21	0.99
Christmas	7.8	2.5	8.3	1.8	4.4	1.8	0.5	2.2	5.1	3.6	4.1	-0.17	1.15	-0.20	0.71	-0.19	0.84
Recife	8.7	2.9	9.8	2.4	4.1	1.8	0.2	2.6	3.6	5.0	4.6	**-0.29**	0.78	-0.26	1.23	**-0.27**	1.10
Salvador	7.6	2.2	5.8	2.1	3.0	1.4	0.3	2.3	4.6	3.6	3.9	**-0.19**	1.16	**-0.21**	0.85	**-0.20**	0.95
São Luís	6.9	2.8	8.4	2.6	4.9	1.9	0.4	2.4	6.3	4.6	5.0	-0.11	1.51	-0.14	0.99	-0.13	1.14
Teresina	9.1	3.1	9.5	1.8	3.7	1.9	0.3	2.4	6.8	2.6	3.6	-0.21	1.68	**-0.29**	0.43	**-0.27**	0.72
North	7.2	1.9	8.3	2.1	4.6	1.0	0.2	1.7	5.3	3.3	3.8	-0.12	1.43	-0.16	0.83	-0.15	1.00
Bethlehem	8.5	3.2	9.3	1.8	7.5	0.7	0.3	1.9	6.8	3.2	4.1	-0.18	1.97	-0.25	0.91	-0.24	1.17
Boa Vista	7.2	1.5	8.1	2.6	4.5	1.5	0.2	1.5	4.2	3.0	3.4	-0.14	0.94	-0.16	0.60	-0.15	0.69
Macapá	7.2	2.3	10.4	2.8	5.7	0.7	0.2	1.4	4.2	2.4	2.9	-0.16	1.15	-0.19	0.62	-0.18	0.77
Manaus	7.9	1.5	12.7	2.3	4.3	0.8	0.2	1.1	5.9	2.4	3.5	-0.16	1.64	-0.23	0.58	-0.21	0.91
Palmas	5.4	0.2	4.2	1.5	3.8	1.0	0.2	2.1	4.5	4.5	4.5	-0.03	1.23	-0.03	1.23	-0.03	1.23
Porto Velho	7.5	2.2	6.0	1.8	3.0	1.1	0.3	2.4	6.5	3.6	4.4	-0.07	1.81	-0.12	0.94	-0.11	1.20
Rio Branco	6.8	2.1	7.3	1.9	3.1	1.1	0.3	1.6	4.9	3.8	4.1	-0.09	1.26	-0.12	0.93	-0.11	1.03
Southeast^a^	10.8	4.2	8.0	3.7	4.6	2.9	0.6	2.9	4.4	5.3	5.0	**-0.35**	0.68	**-0.33**	0.95	**-0.34**	0.87
Belo Horizonte	9.8	3.7	4.9	2.3	3.9	2.3	0.3	2.9	4.0	4.9	4.6	**-0.27**	0.77	**-0.25**	1.06	**-0.26**	0.97
Rio de Janeiro	13.3	5.6	10.6	6.5	5.5	4.3	0.6	2.9	4.3	5.5	5.2	**-0.43**	0.23	**-0.41**	0.59	**-0.41**	0.49
São Paulo	11.3	3.6	8.2	2.2	4.2	2.2	0.6	3.0	4.2	4.7	4.6	**-0.43**	0.83	**-0.42**	1.01	**-0.42**	0.96
Victoria	9.0	3.6	8.0	3.8	4.7	2.9	0.8	2.9	5.2	6.1	5.7	**-0.26**	0.88	**-0.25**	1.15	**-0.25**	1.05
Central-West^a^	9.3	3.6	7.2	3.6	3.4	2.1	0.4	2.6	6.5	4.5	5.1	-0.14	1.53	-0.18	0.93	-0.17	1.11
Campo Grande	7.0	0.9	8.3	1.7	3.2	2.6	0.3	2.7	7.9	2.9	4.4	-0.05	1.83	-0.15	0.31	-0.12	0.76
Cuiabá	7.7	2.4	6.8	3.2	3.2	2.3	0.2	2.5	9.2	5.6	6.7	0.00	2.30	-0.07	1.23	-0.05	1.55
Federal District	13.8	7.2	7.5	5.3	3.5	1.9	0.6	2.5	4.9	4.7	4.8	-0.30	1.10	-0.30	1.03	-0.30	1.06
Goiânia	9.0	3.9	6.1	4.3	3.5	1.7	0.5	2.6	3.9	4.8	4.5	**-0.22**	0.89	**-0.20**	1.14	**-0.21**	1.07
South	12.2	3.1	11.5	4.0	5.9	3.1	0.7	2.8	4.8	4.7	4.7	-0.28	0.71	-0.28	0.69	-0.28	0.67
Curitiba	8.9	2.8	10.0	2.9	4.4	2.3	0.4	2.8	5.0	4.8	4.9	-0.18	1.04	-0.18	1.00	-0.18	1.01
Florianópolis	12.7	4.3	10.9	5.4	6.8	3.4	0.9	2.8	5.3	5.0	5.1	**-0.32**	0.77	**-0.33**	0.65	**-0.32**	0.69
Porto Alegre	15.0	2.0	13.5	3.7	6.7	3.6	0.9	2.9	4.0	4.3	4.2	-0.35	0.32	-0.34	0.40	-0.34	0.38
Total^b^	9.1	3.0	8.7	2.8	4.4	1.9	0.4	2.4	5.3	4.1	4.5	-0.21	1.19	**-0.23**	0.86	**-0.23**	0.95

^a^ Average values for the capitals of each region.

^b^ Average values for the 26 capitals and the Federal District.

^c^ Data only for the landline register in 2023.

^d^ Data only for the mobile phone register in 2023.

^e^ Combined data from the landline and mobile phone register in 2023.

*Global refusal, involving both refusals related to the interview and those related to the appointment (more information in the Methods section).

Statistically significant values are shown in bold (p < 0.05).

### Days and Times when Contacts are Most Frequent

**Figure 1:**
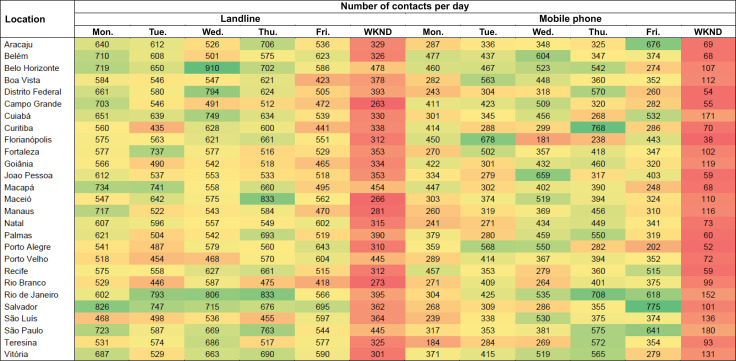
Number of contacts answered in Brazilian state capitals and the Federal District, by day of the week for landline and mobile phone registration. Vigitel Brasil, 2023.

**Figure 2:**
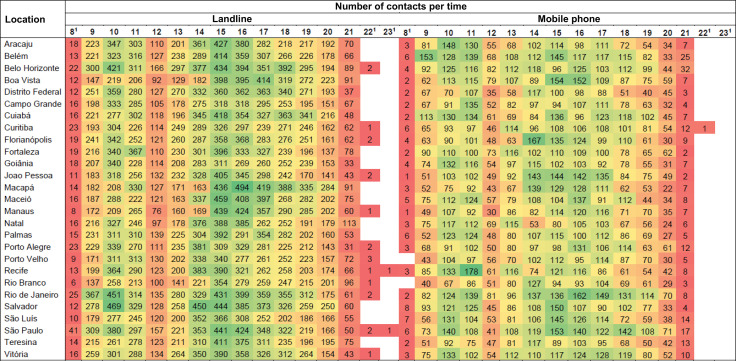
Number of contacts answered in Brazilian state capitals and the Federal District, by time of day for landline and mobile phone registration. Vigitel Brasil, 2023.

^1^ Contact outside office hours is only made at the express request of the interviewee.

Note: the cooler (greenish) colors on the scale indicate the times of day with the highest number of contacts, while the warmer (reddish) areas indicate those with the lowest number of contacts. The data does not consider the difference in the distribution of interviewers between times of day. A total of 60 interviewers took part in the operation, 40 from 8:45 to 15:15 and 20 from 15:15 to 21:30. At weekends, a single shift of interviewers worked from 9:20 to 15:40.

In general, in 2023, Vigitel interviews were more concentrated on weekdays than on weekends, for both landlines and mobile phones (Figure 1). Considering the time of the calls, the highest number of contacts answered was between 9am and 8pm for both landlines and mobile phones (Figure 2). Data on the number of contacts answered is also described by day of the week (Figure 1) and time of day (Figure 2).

FDS: contact on weekends, Saturdays and Sundays, and public holidays.

Note: The cooler (greenish) colors on the scale indicate the days of the week with the highest number of contacts, while the warmer (reddish) colors indicate those with the lowest number of contacts. The data does not consider the difference in the distribution of interviewers between periods of the day. A total of 60 interviewers took part in the operation, 40 from 8:45 to 15:15 and 20 from 15:15 to 21:30. At weekends, a single shift of interviewers worked from 9:20 to 15:40.

## DISCUSSION

This study analyzed the trend of Vigitel’s operational indicators over its 17 editions, revealing a challenging scenario. The eligibility rate fell over the entire period, and more sharply in recent years, when the reduction was almost four times that of the total period. Although the inclusion of mobile phones in the register in 2023 eased the drop in eligibility, the reduction still remained high. The response rate also fell over the whole period and, together with the fall in eligibility, represents an obstacle to the sustainability of the survey. On the other hand, the refusal rate fell over the whole period, showing the consistent quality of the system’s operation. In this context, our study provides an up-to-date and comprehensive analysis of important methodological issues for improving the quality and continuity of Vigitel.

Eligibility rates have fallen sharply since the start of the system, with values between 60% and 70% representing the trend between 2007 and 2017, followed by a sharp decline. The occurrence of ineligible lines, especially due to registration lags, should be accepted as a natural phenomenon, since the lines to be called throughout the operation are drawn at a single point in time. However, the intense reduction in the eligibility rate, from 72.5% to 5.3% in 2023 for landlines, exposes an important weakness of telephone surveys with a single data collection frame.

In Vigitel 2023, 61.3% of landlines and 54.5% of ineligible mobile lines corresponded to lines for which contact could not be made after six or more contact attempts on different days and at different times (data not shown). It should be noted that the methodology used to calculate the Vigitel rates (AAPOR #2) treats as ineligible those lines for which it was not possible to definitively identify their eligibility^16^. This increase in the number of ineligible lines coupled with low coverage can reduce the quality of the data collected, especially in regions with reduced coverage, such as the Northeast and North. These problems also have an impact on the cost of the survey, increasing the number of calls and screening attempts^18,19^.

It should be noted that even with the large drops seen in recent years, Vigitel has eligibility rates higher than those of the Behavioral Risk Factor Surveillance System (BRFSS), conducted by the Centers for Disease Control and Prevention (CDC) in the United States^20^. It should be noted, however, that the calculation of the eligibility rates applied in these surveys differs from one another. In Vigitel (AAPOR #2), individuals who answered the survey or not make up the denominator of the calculation. In the BRFSS (AAPOR #4), only those who did not complete the survey are included in the eligibility calculation, excluding those who did^16^. These methodological differences limit the comparability of these surveys.

Some factors are related to these results, such as the rapid increase in the use of cell phones and the Internet by the population^18^, also suggesting that telephone interviews (by voice) should coexist with Internet interviews (by data)^21^. Thus, some surveys are migrating to cell phones, the Internet, or adopting mixed modalities to compensate for the lack of landline coverage and non-response^19,22^. It is worth noting, however, that around 40% of ineligible lines fall into the categories of business or commercial line, out of service, non-existent, and empty holiday or vacation home, which would be more related to weaknesses in the quality of the register than to social movements beyond the control of the survey.

It is widely recognized that response rates in telephone surveys are decreasing over time, as “non-response” increases^18^. In this study, we found lower response rates for mobile phones compared to landlines. Vigitel 2023 was the first edition to include mobile phones in its register. Since 1984, the BRFSS has been considered the largest telephone survey system in the world. Since 2011, the BRFSS has adopted the use of cell phone interviews, complementing landline interviews, which has improved the validity, quality and representativeness of the survey. The survey scenario is even less favorable than Vigitel’s, since in 2022 the combined response rate (mobile + landline) was 45.1%^23^. Since mobile phones are equipped with caller ID and electronic processes to block unwanted calls, only interviews concluded on the first contact are usually successful, with a tiny proportion of appointments being converted into success^19^.

For 2023, the scenario identified suggests a reduction in the quality of the operation, possibly due to the short time available for implementation, the remote format of the collection, the gradual incorporation of new interviewers, the poor quality of the register used and the short period for collecting a significant volume of interviews (data collection took place over around four months - between December 2022 and April 2023). While the 2020 operations had around 40 interviewers, the 2023 operation required around 60.

Although telephone contact has become more difficult, considering the eligibility and success rates, Vigitel’s refusal rate is possibly the indicator that best expresses the quality of the system’s operation, and this indicator has evolved favorably, decreasing over the years. It is worth noting that in 13 of the 17 years under study, the refusal rate was below 5%, a very significant figure for surveys of this nature. The figures presented for the refusal rate refer both to refusal to schedule (obtained during the initial contact with the household, which does not even allow a resident to be drawn) and to refusal to take part in the interview (when a resident of the household has already been drawn and refuses to take part in the study). Contrary to these results, studies have shown an increase in refusal rates in telephone surveys, which is strongly associated with the initial screening of the interviewer^18,19^. Thus, the decrease in the refusal rate reveals the interviewer’s priority in obtaining the interviewee’s cooperation, which may be related to the experience acquired over more than a decade of interviewing, especially through interviewer training and supervision^13^.

An analysis of the Vigitel indicators allows us to conclude that the methodology used has become fatigued, with a reduction in eligibility and response rates. This is a common occurrence in population surveys, which must adapt periodically to social and technological changes^21^. At the moment, only the refusal rate has been saved, which, despite being far from its lowest levels, remains at satisfactory levels. It is noticeable that telephone surveys as the only method of data collection have become less convenient as exchanges of information via data (such as short message services - SMS and automatic messaging platforms) become more frequent^21^. The search for alternatives that make participation more convenient for the respondent is undoubtedly a constant path for surveys of this nature.

Based on these results, it is not possible to infer the magnitude of influence of each of these factors on the overall quality of the survey, and this conclusion is dependent on the occurrence of a new Vigitel operation under ideal planning and execution conditions, as happened in previous years^13^. It is also believed that the survey will benefit from the inclusion of a new means of collection, so the continuity of studies to keep up with the current technological dynamism will be important for maintaining the survey.

It is important to highlight the consolidation of VIGITEL as a surveillance system for risk and protective factors for CNCDs in Brazil, setting an example of a data collection initiative with good quality, low cost, and great agility, a pioneer in middle- and low-income countries. It should be emphasized that the sustainability of the data collection structure is a fundamental step in taking the system back in the right direction, as well as the continuity of studies capable of pointing out and incorporating improvements to its methodology.
