# Supplementary material for: Time trend of Vigitel Brasil operation indicators (2006 to 2023)
Source: Rev Saude Publica. 2025 Jun 11;59:e20. doi: 10.11606/s1518-8787.2025059006369 (PMC12211791; doi:10.11606/s1518-8787.2025059006369)
Supplement: Translation

# Translation: Tendência temporal de indicadores da operação do Vigitel Brasil (2006 a 2023)

**Authors:** Taciana Maia de Sousa, Luiza Eunice Sá da Silva, Laura Cordeiro Rodrigues, Thaís Cristina Marquezine Caldeira, Letícia de Oliveira Cardoso, Rafael Moreira Claro

**Affiliations:** I Brasil Universidade do Estado do Rio de Janeiro. Instituto de Nutrição. Departamento de Nutrição Social. Rio de Janeiro, RJ, Brasil; II Brasil Universidade Federal de Pelotas. Faculdade de Medicina. Pelotas, RS, Brasil; III Brasil Universidade Federal de Minas Gerais. Faculdade de Medicina. Departamento de Medicina Preventiva e Social. Belo Horizonte, MG, Brasil; IV Brasil Ministério da Saúde. Secretaria de Vigilância em Saúde e Ambiente. Departamento de Análises Epidemiológicas e Vigilância de Doenças e Agravos Não Transmissíveis. Brasília, DF, Brasil; V Brasil Universidade Federal de Minas Gerais. Escola de Enfermagem. Departamento de Nutrição. Belo Horizonte, MG, Brasil

**Keywords:** Estudo Metodológico, Inquérito de Saúde, Vigilância em Saúde Pública, Fatores de Risco, Brasil

## Abstract

**OBJETIVO:**

Analisar a tendência temporal de indicadores da operação de coleta dos dados do Vigitel entre 2006 e 2023.

**MÉTODOS:**

Estudo metodológico que analisou a tendência temporal de três indicadores da operação do Vigitel: taxa de elegibilidade, taxa de resposta e taxa de recusa, além do número de contatos atendidos segundo dias da semana e horários. Uma série de regressões lineares foi empregada para identificar variações temporais para o período completo (2006 a 2023) e para o quinquênio final desse período (2018 a 2023).

**RESULTADOS:**

Durante as 17 edições analisadas, observou-se uma diminuição na taxa de elegibilidade (-3,26 pp/ano; p < 0,001) com maior magnitude no período mais recente (-12,23 pp/ano; p = 0,010). Embora a inclusão de telefones móveis no cadastro em 2023 tenha atenuado a queda da elegibilidade, sobretudo nos últimos anos, a redução permaneceu elevada (-10,43 pp/ano; p = 0,020). A taxa de resposta também diminuiu no período completo (-0,78; p < 0,001) e, conjuntamente com a queda da elegibilidade, representa um obstáculo para a sustentabilidade do inquérito. Em contrapartida, a taxa de recusa também diminuiu (-0,23 pp/ano; p = 0,030), indicando a consistente qualidade na operação do sistema. O número de contatos atendidos se concentrou em dias da semana e horário entre 9h e 20h.

**CONCLUSÕES:**

A análise dos dados da operação do inquérito Vigitel sugere uma fadiga da metodologia empregada, com reduções na elegibilidade e na taxa de resposta ao longo dos anos. Apenas a taxa de recusa que, a despeito de estar longe de seus menores níveis, permanece em patamares satisfatórios. A busca de alternativas que tornem a participação mais conveniente para o respondente é um caminho importante para inquéritos dessa natureza, visando a manutenção da qualidade da vigilância em saúde no País.

## INTRODUÇÃO

Aprimorar os sistemas de saúde global requer a avaliação contínua de dados sólidos e de qualidade, obtidos por meio de sistemas de informação e inquéritos de saúde atualizados para refletir com precisão as condições de saúde da população^1^. Nas últimas décadas, mediante esforços de organizações internacionais, governos e instituições de pesquisa, o uso de inquéritos de saúde em países de renda baixa e média foi ampliado^1^. No Brasil, principalmente a partir da década de 1980, dados primários passaram a ser coletados por meio de inquéritos populacionais reconhecidos como componentes fundamentais de um sistema nacional de informações em saúde^3^.

Entre os inquéritos de saúde brasileiros, o Sistema de Vigilância de Fatores de Risco e Proteção para Doenças Crônicas por Inquérito Telefônico (Vigitel) se destaca pela sua realização desde 2006, abrangendo uma amostra representativa da população adulta (≥ 18 anos) residente em todas as capitais dos 26 estados brasileiros e no Distrito Federal^6^. A concepção do Vigitel no início dos anos 2000 foi impulsionada pelo panorama epidemiológico da época, quando análises de séries históricas de mortalidade nas capitais dos estados brasileiros indicavam aumento de mais de três vezes na proporção de mortes por doenças crônicas não transmissíveis (DCNT) entre 1930 e 2006^7^.

Desde então, em 17 edições, o Vigitel tem sido o inquérito responsável por monitorar, de forma contínua, a tendência temporal na prevalência das DCNT e seus principais fatores de risco em capitais brasileiras. Os dados coletados anualmente permanecem como indicador sólido da evolução dos indicadores de saúde, essenciais para o monitoramento das ações de prevenção e controle das DCNT, como o Plano de Ações Estratégicas para o Enfrentamento das Doenças Crônicas Não Transmissíveis no Brasil, 2011 a 2022^8^, e sua versão mais atual, 2021 e 2030^9^.

Para fornecer um panorama acurado da situação de saúde do Brasil, é crucial que o Vigitel seja metodologicamente revisado com base na observação de possível queda de qualidade da informação gerada. Cabe destacar que, desde sua concepção, o Vigitel permaneceu com metodologia de amostragem e coleta dos dados muito semelhante àquela adotada em sua primeira edição, indicando sua robustez ao longo do tempo. De 2006 a 2021, as entrevistas foram conduzidas exclusivamente por telefone fixo^10^. Contudo, a progressiva redução da cobertura de telefonia fixa no País, especialmente nas capitais das regiões Norte e Nordeste, apontavam a necessidade iminente de incorporar um novo modal de coleta de dados ao Vigitel^11^.

Os dados de pesquisas domiciliares no Brasil já apontavam para essa deterioração da cobertura de telefonia fixa. Em 2019, somente 30,4% dos domicílios nas capitais brasileiras tinham telefone fixo, apresentando uma expressiva redução em relação ao ano de 2013 (48,9%)^12^. Esses resultados também evidenciaram disparidade na cobertura entre as capitais, com apenas 9,2% em Macapá e 53,2% no Rio de Janeiro^12^. Estudo anterior relatou a diminuição na taxa de elegibilidade das linhas de telefonia fixa no País^13^, tornando inviável a realização do inquérito exclusivamente por esse modal. Como resposta a essa situação, em 2023, o Ministério da Saúde (MS) incorporou entrevistas por telefone móvel ao inquérito. Contudo, até o momento não foram conduzidos estudos que oferecessem uma análise abrangente da operação do Vigitel.

Assim, o presente estudo teve como objetivo analisar a tendência temporal dos principais indicadores da operação de coleta dos dados do Vigitel, no período entre 2006 e 2023.

## MÉTODOS

Trata-se de estudo metodológico desenvolvido a partir de informações registradas durante a operação do Vigitel entre 2006 e 2023. Todas as informações analisadas foram fornecidas pela empresa contratada responsável pela coleta dos dados e enviada semanalmente para o MS para fins de acompanhamento da evolução do inquérito ano longo dos anos.

### Contextualização da Amostragem e Coleta de Dados do Vigitel

Desde 2006, o Vigitel entrevista anualmente aproximadamente 54 mil indivíduos com 18 anos ou mais, distribuídos em cerca de duas mil entrevistas em cada uma das capitais dos 26 estados brasileiros e no Distrito Federal. Contudo, a partir de 2020, devido à pandemia da covid-19, foi implementado um expediente emergencial, reduzindo a amostra para mil indivíduos em cada local^10,14^. Em 2023, nova redução se fez necessária devido a questões administrativas e operacionais, com amostra de cerca de oitocentos indivíduos por capital^6^.

O processo de amostragem do inquérito é realizado em duas etapas. Na primeira, um número predeterminado de 10.000 linhas telefônicas é sorteado em cada cidade. Em seguida, as linhas selecionadas em cada cidade são ressorteadas em réplicas de 200 linhas. Com isso, cada réplica deve representar proporção semelhante de linhas por região da cidade ou prefixo telefônico àquela do conjunto total da amostra. Resultados de estudos-piloto realizados anteriormente com listagens de telefone móvel obtidas por RDD (discagem aleatória de dígitos) indicaram a baixa qualidade dos cadastros de telefone móvel. Portanto, optou-se por réplicas com 500 linhas para esse modal.

A segunda etapa começa com a identificação das linhas sorteadas que são elegíveis para o sistema, ou seja, linhas residenciais ativas (metodologia aplicada entre 2006 e 2021). Não foram consideradas elegíveis para o sistema as linhas atribuídas a empresas, as que não estão em uso ou que estão fora de serviço. Além disso, as linhas que não respondem a seis chamadas (entre 2006 e 2008 eram realizadas até 10 chamadas), realizadas em diferentes dias e horários, incluindo sábados e domingos e períodos noturnos, e que provavelmente correspondem a domicílios desocupados, também foram consideradas inelegíveis.

Para cada linha fixa elegível, uma vez obtida a concordância do respondente em participar do inquérito, os moradores adultos do domicílio são enumerados e, em seguida, é realizado um sorteio aleatório para selecionar um desses adultos para a entrevista. Caso a pessoa sorteada se encontre presente e disponível, a entrevista é realizada no contato inicial. Caso contrário, a entrevista poderia ser agendada para dia e horário mais conveniente. A recusa em participar do estudo em cada um desses dois momentos é registrada e somada para compor o indicador de recusa quanto à participação no estudo.

Para a operação com telefones móveis (adotada a partir de 2023) foi implementada metodologia semelhante, sem a necessidade do sorteio intradomiciliar após a confirmação da elegibilidade. Tal procedimento é necessário no caso do telefone fixo, uma vez que a linha tem propriedade atrelada ao domicílio, mas é dispensável nos telefones móveis uma vez que sua propriedade tende a ser individual e seu uso não é compartilhado entre os membros da família^15^.

### Indicadores de Qualidade do Cadastro e da Performance do Sistema

Ao longo de suas edições, o Vigitel acompanhou os seguintes indicadores de qualidade do Sistema: 1) Taxa de elegibilidade: percentual de linhas consideradas elegíveis para o Vigitel entre o conjunto completo das linhas sorteadas; 2) Taxa de resposta: percentual de entrevistas efetivamente realizadas entre as linhas sorteadas consideradas elegíveis; e 3) Taxa de recusa: percentual de linhas elegíveis que recusaram participação no estudo, incluindo tanto recusas de entrevista quanto recusas de agendamento).

Uma vez que metodologias distintas para o cálculo dessas taxas em inquéritos telefônicos se fazem disponíveis, no Vigitel, desde 2006, optou-se por empregar a metodologia padrão *American Association for Public Research* (AAPOR) #2. Segundo essa metodologia, as taxas de sucesso e recusa são estimadas considerando apenas as linhas identificadas como elegíveis, e que linhas com *status* indefinidos são assumidas como inelegíveis^16^.

Adicionalmente, incluem-se informações relativas aos dias e horários de maior efetividade na operação de 2023. Um total de 60 entrevistadores participou da operação, sendo 40 no período 8h45 às 15h15 e 20 no período das 15h15 às 21h30. Aos finais de semana, um turno único de entrevistadores trabalhava das 9h20 às 15h40.

### Organização e Análise de Dados

Todos os dados foram organizados e analisados por meio do *software* Stata^®^ versão 16.0^17^.

Inicialmente, dados relativos aos três indicadores de qualidade do sistema, além do número de contatos atendidos segundo dias da semana e horários, apresentados na seção anterior, foram estimados como base nos microdados da operação fornecidos pela empresa responsável pela coleta dos dados. Essa operação foi conduzida para cada uma das cidades, para o conjunto das cidades de uma mesma região geográfica (Norte, Nordeste, Centro-Oeste, Sul e Sudeste) e para o conjunto completo das cidades estudadas pelo Vigitel.

Posteriormente, uma série de regressões lineares foi empregada com o objetivo de identificar variações significativas no período estudado. Em cada modelos de regressão, o indicador de qualidade do Vigitel em questão foi considerado como variável desfecho, enquanto o ano de realização do inquérito foi tratado como variável de exposição. Nesse tipo de análise, o coeficiente angular do modelo de regressão (valor Beta) indica a variação média do indicador em pontos percentuais ao ano (pp/ano) e o valor de P identifica se essa variação foi estatisticamente significativa (p < 0,05). Essa análise de variação foi conduzida para o período completo de realização do Vigitel (de 2006 a 2023) e para o quinquênio final desse período (de 2018 a 2023). Adicionalmente, foi realizada análise de variação para um recorte do período completo, excluindo-se os anos de 2006, 2007 e 2008 (material suplementar^a^), devido às mudanças ocorridas na operação do inquérito em seus anos iniciais, que poderiam enviesar a comparação no conjunto completo do período. Ressalta-se que não há evidência de erro nos dados para esse período, no entanto, até 2009 as taxas eram calculadas manualmente, passando a serem calculadas de forma automática após aquele momento.

Dada a inclusão de telefones móveis no inquérito em 2023, foram realizadas três análises adicionais. A primeira considerou apenas o quadro de telefones fixos do Vigitel 2023, a segunda, somente o quadro de telefones móveis e a terceira unificou fixos e móveis em um quadro síntese.

Por fim, os dados referentes aos dias e horários de maior realização de entrevistas foram organizados na forma de tabelas contendo mapas de calor. Novamente, as apresentações foram divididas entre quadro para telefones fixos e móveis. Esses valores foram tabulados de forma bruta, sem considerar a variação no número de entrevistadores nos diferentes dias e horários, pois o número e a distribuição dos entrevistadores não foram constantes durante a operação. A operação do Vigitel 2023 teve início com seis entrevistadores, aumentando gradativamente até os 60 entrevistadores durante os primeiros 45 dias de coleta. A expectativa inicial envolvia contar com turnos de tamanho semelhantes quanto ao número de entrevistadores. No entanto, devido à baixa conversão de entrevistas por telefone fixo, indicou-se a possibilidade de redução dos trabalhadores no segundo turno, possibilitando a redução dos custos da operação. Portanto, na respectiva seção, também foram apresentados o número e a distribuição de entrevistadores no período de fevereiro a abril de 2023.

## RESULTADOS

### Taxa de Elegibilidade

Durante o período completo estudado, a taxa de elegibilidade das linhas de telefone fixo sorteadas para inclusão no Vigitel variou entre 72,5% e 5,3%. Ao considerar apenas a operação de 2023, o cadastro de telefonia móvel apresentou uma taxa de elegibilidade quase três vezes superior àquela do cadastro de telefonia fixa (21,2% *versus* 5,3%). A composição de uma estimativa envolvendo ambos os cadastros, resultou em uma taxa de elegibilidade de 11,3%, mitigando a forte queda observada nos últimos anos (Tabela 1).

**Tabela 1:** Percentual (%) de linhas telefônicas elegíveis nas capitais dos estados brasileiros e no Distrito Federal identificado a partir do total de linhas sorteadas para operação do sistema, por ano. Vigitel Brasil, 2006 a 2023.

Localidade	Ano											Variação média no período (pp/ano)					
	2006	2009	2012	2015	2018	2019	2020	2021	2023^c^	2023^d^	2023^e^	2006/23^c^	2018–2023^c^	2006–2023^d^	2018–2023^d^	2006–2023^e^	2018–2023^e^
Nordeste^a^	81,3	65,5	63,1	69,7	45,0	44,0	35,0	14,6	6,7	20,8	12,8	**-3,58**	**-13,22**	**-3,30**	-9,01	**-3,46**	**-11,40**
Aracaju	88,9	67,3	61,9	70,8	45,5	45,2	36,2	16,2	6,6	25,2	14,0	**-3,76**	**-13,60**	**-3,40**	-8,02	**-3,62**	**-11,37**
Fortaleza	84,7	65,8	68,8	71,4	50,8	46,1	41,0	19,8	7,7	17,2	12,6	**-3,43**	**-13,65**	**-3,24**	-10,78	**-3,33**	**-12,16**
João Pessoa	78,8	67,4	62,3	74,8	35,6	38,8	24,4	7,2	4,8	21,4	11,0	**-4,07**	**-11,92**	**-3,74**	-6,94	**-3,95**	-10,06
Maceió	77,4	63,5	60,2	74,0	44,3	42,9	32,9	9,9	7,6	16,8	11,9	**-3,56**	**-12,89**	**-3,38**	-10,15	**-3,48**	-11,61
Natal	80,7	65,5	65,8	64,8	35,6	43,4	34,1	12,4	7,0	17,3	12,1	**-3,70**	**-13,08**	**-3,49**	-9,99	**-3,60**	-11,56
Recife	80,9	65,7	64,0	65,5	49,1	47,9	42,3	17,7	5,9	26,7	13,5	**-3,39**	**-15,06**	**-2,98**	-8,83	**-3,24**	**-12,79**
Salvador	81,5	63,2	69,8	74,5	57,3	50,9	45,1	18,8	8,7	18,8	13,9	**-3,10**	**-15,31**	**-2,91**	-12,27	**-3,00**	**-13,75**
São Luís	76,5	64,1	54,6	60,4	40,4	38,9	27,2	12,6	6,5	21,6	13,2	**-3,64**	**-11,17**	**-3,35**	-6,63	**-3,51**	-9,16
Teresina	82,3	67,2	60,4	71,4	46,2	41,7	32,1	17,1	5,7	21,9	13,1	**-3,54**	**-12,31**	**-3,23**	-7,44	**-3,40**	**-10,10**
Norte^a^	68,6	59,4	54,7	61,2	33,8	26,8	18,8	9,9	3,7	21,6	8,7	**-3,52**	**-7,84**	**-3,17**	-2,45	**-3,42**	**-6,32**
Belém	84,6	66,0	55,8	55,4	43,2	36,3	35,2	13,6	5,4	26,0	13,3	**-3,71**	-11,42	**-3,30**	-5,25	**-3,55**	-9,07
Boa Vista	78,7	60,4	55,2	60,1	33,7	20,1	13,0	9,0	2,2	22,8	6,3	**-4,32**	**-5,78**	**-3,92**	0,39	**-4,24**	**-4,56**
Macapá	68,7	62,2	53,5	59,6	31,4	21,8	10,2	8,7	2,4	19,0	6,2	**-4,03**	-5,98	**-3,70**	-1,01	**-3,95**	-4,83
Manaus	78,3	61,8	49,6	57,2	30,1	26,1	19,4	6,9	3,4	20,8	8,0	**-4,10**	**-8,07**	**-3,76**	-2,84	**-4,01**	-6,70
Palmas	52,3	54,6	60,9	62,7	26,6	23,3	12,7	8,3	3,8	20,9	8,7	**-2,81**	**-6,27**	**-2,47**	-1,15	**-2,71**	-4,81
Porto Velho	58,1	55,3	58,4	67,7	36,5	29,9	21,3	11,7	3,7	21,9	8,7	**-2,81**	**-8,84**	**-2,45**	-3,37	**-2,71**	**-7,31**
Rio Branco	59,8	55,5	49,8	65,4	35,2	30,1	20,2	11,0	4,9	20,1	10,0	**-2,86**	**-8,50**	**-2,56**	-3,92	**-2,76**	**-6,94**
Sudeste^a^	77,8	50,1	66,8	55,7	55,5	49,5	44,2	22,8	7,8	23,1	14,4	**-2,86**	**-14,64**	**-2,55**	-10,04	**-2,73**	**-12,65**
Belo Horizonte	87,7	71,0	69,7	73,7	62,1	53,3	46,7	26,8	8,8	19,8	14,2	**-3,16**	**-15,33**	**-2,94**	**-12,04**	**-3,05**	**-13,73**
Rio de Janeiro	77,4	63,8	67,3	72,9	58,9	50,4	43,2	19,3	6,7	26,4	14,6	**-3,00**	**-15,49**	**-2,61**	-9,59	**-2,84**	**-13,13**
São Paulo	72,1	57,2	63,5	67,4	56,5	46,0	43,7	22,6	8,5	25,7	16,8	-2,10	**-13,38**	-1,77	-8,21	-1,94	-10,88
Vitória	74,2	65,0	66,7	75,4	44,5	48,1	43,0	22,4	7,1	20,7	12,1	**-3,16**	**-14,36**	**-2,90**	-10,31	**-3,06**	**-12,86**
Centro-oeste^a^	60,8	53,1	60,9	64,9	45,6	43,6	36,0	18,9	6,6	19,5	11,9	**-1,95**	**-12,81**	**-1,70**	-8,96	**-1,85**	**-11,22**
Campo Grande	64,1	50,8	56,3	61,2	39,0	41,4	30,7	17,6	4,6	17,7	9,6	**-2,08**	**-12,37**	**-1,82**	-8,42	**-1,98**	**-10,87**
Cuiabá	56,4	53,9	56,3	65,4	32,9	37,7	28,0	16,0	4,6	22,2	10,3	**-2,21**	**-11,13**	**-1,87**	-5,85	**-2,10**	**-9,41**
Distrito Federal	62,9	50,4	65,1	68,5	57,5	45,7	41,5	18,8	8,8	18,8	13,8	-1,74	**-13,35**	-1,54	-10,35	-1,64	**-11,86**
Goiânia	59,8	57,1	66,1	64,5	53,1	49,7	43,7	23,2	8,6	19,1	14,0	**-1,76**	**-14,37**	**-1,55**	**-11,21**	**-1,65**	**-12,74**
Sul^a^	63,3	54,1	63,4	64,4	49,4	45,3	39,4	20,6	7,5	23,1	14,6	**-1,95**	**-13,22**	**-1,65**	-8,55	**-1,81**	**-11,08**
Curitiba	64,9	57,9	65,2	66,9	55,2	49,7	44,9	24,0	7,8	22,6	15,0	**-1,85**	**-14,69**	**-1,56**	-10,23	**-1,71**	**-12,53**
Florianópolis	63,5	53,2	64,2	63,8	41,2	42,2	36,5	19,1	7,5	21,8	13,9	**-2,01**	**-12,15**	**-1,74**	-7,87	**-1,89**	**-10,23**
Porto Alegre	61,6	51,1	60,8	62,4	52,0	43,9	36,8	18,6	7,2	24,8	15,0	**-1,99**	**-12,82**	**-1,64**	-7,55	**-1,83**	**-10,48**
Total^b^	72,5	58,5	61,2	64,1	44,2	40,4	32,8	15,9	5,3	21,2	11,3	**-3,26**	**-12,23**	**-2,95**	-7,45	**-3,14**	**-10,43**

^a^ Valores médios das capitais de cada região.

^b^ Valores médios das 26 capitais e do Distrito Federal.

^c^ Dados apenas para o cadastro de telefonia fixa em 2023.

^d^ Dados apenas para o cadastro de telefonia móvel em 2023.

^e^ Dados combinados do cadastro de telefonia fixa e móvel em 2023.

Valores com significância estatística estão apresentados em negrito (p < 0,05).

Considerando apenas o cadastro de telefones fixos para a operação 2023, observou-se redução média da taxa de elegibilidade de 3,26 pp/ano (p < 0,001) no período entre 2006 e 2023. No entanto, a redução observada no período mais recente (2018 a 2023) foi mais de três vezes superior (12,23 pp/ano; p = 0,010). Quando são considerados ambos os cadastros, a redução no período mais recente foi de 10,43 pp/ano (p = 0,020). Embora menor que no caso dos telefones fixos, ainda é um valor significativamente elevado (Tabela 1).

Considerando ainda o cadastro de linhas fixas, para todo o período, foram observadas reduções, principalmente, para as capitais das regiões Nordeste (-3,58 pp/ano; p < 0,001) e Norte (-3,52 pp/ano; p < 0,001) do País. No período mais recente, maior magnitude de redução foi observada nas capitais da região Sudeste (-14,64 pp/ano; p = 0,010) (Tabela 1).

### Taxa de Resposta

No conjunto completo do período, a taxa de resposta variou de 71,5% em 2006 a 58,7% em 2023, quando considerados apenas o cadastro de telefones fixos (-0,78pp/ano; p < 0,001). Para o ano de 2023, o cadastro de telefones móveis apresentou taxa de resposta cerca de 60% inferior àquela do cadastro de fixos (24,1% *versus* 58,7%) (Tabela 2).

**Tabela 2:** Percentual (%) de resposta dentre as linhas telefônicas elegíveis sorteadas nas capitais dos estados brasileiros e no Distrito Federal, por ano. Vigitel Brasil, 2006 a 2023.

Localidade	Ano											Variação média no período (pp)					
	2006	2009	2012	2015	2018	2019	2020	2021	2023^c^	2023^d^	2023^e^	2006–2023^c^	2018–2023^c^	2006–2023^d^	2018–2023^d^	2006–2023^e^	2018–2023^e^
Nordeste^a^	71,2	74,8	64,4	70,7	70,7	70,7	57,6	60,7	57,9	23,3	33,1	**-0,71**	-3,54	**-1,39**	-13,94	**-1,20**	-11,00
Aracaju	67,3	74,9	64,5	70,8	71,0	73,2	58,2	59,5	56,8	22,2	32,0	-0,60	-4,80	**-1,27**	-15,16	**-1,08**	-12,24
Fortaleza	70,1	72,8	64,5	70,1	70,4	74,2	58,3	60,6	60,7	25,3	35,7	**-0,57**	-3,82	**-1,26**	-14,45	**-1,06**	-11,32
João Pessoa	71,0	74,5	66,9	70,3	72,1	68,2	58,6	70,7	57,9	21,8	31,6	**-0,57**	-1,90	**-1,28**	-12,73	**-1,08**	-9,78
Maceió	72,5	75,4	65,8	71,3	70,2	68,7	54,3	56,2	54,1	28,1	37,0	**-0,92**	-4,20	**-1,43**	-11,99	**-1,26**	-9,33
Natal	73,4	77,3	64,5	70,7	70,1	68,4	58,9	63,4	57,5	23,9	33,8	**-0,80**	-2,81	**-1,46**	-12,89	**-1,27**	-9,92
Recife	73,2	72,8	65,3	69,7	70,1	73,9	59,3	61,4	59,1	23,0	33,0	**-0,65**	-4,22	**-1,36**	-15,06	**-1,16**	-12,04
Salvador	68,5	76,1	63,5	70,8	70,9	74,8	53,5	55,5	51,3	22,4	31,2	**-0,96**	-6,83	**-1,53**	-15,51	**-1,35**	-12,87
São Luís	73,0	74,7	61,3	72,2	70,9	68,1	54,7	57,5	57,9	22,0	31,8	**-0,92**	-2,79	**-1,62**	-13,57	**-1,43**	-10,62
Teresina	71,8	74,8	62,8	70,1	70,4	67,2	62,9	61,2	66,2	20,8	31,5	-0,42	-0,48	**-1,31**	-14,11	**-1,10**	-10,89
Norte^a^	74,3	77,1	65,3	70,8	71,0	64,8	53,4	63,0	56,3	23,9	33,5	**-1,05**	-1,57	**-1,68**	-11,28	**-1,49**	-8,41
Belém	70,3	76,2	60,8	72,3	72,3	66,0	53,1	56,4	53,1	17,6	26,6	**-0,99**	-3,54	**-1,68**	-14,18	**-1,51**	-11,50
Boa Vista	71,3	75,7	61,4	70,3	74,1	64,7	51,1	61,3	57,7	22,8	32,7	**-0,85**	-1,09	**-1,53**	-11,56	**-1,34**	-8,59
Macapá	73,3	73,5	61,6	70,3	66,4	58,7	54,1	56,8	53,5	22,5	31,6	-1,45	-1,29	**-2,06**	-10,58	**-1,88**	-7,86
Manaus	71,5	74,3	65,7	70,1	72,0	66,7	50,9	70,0	55,0	25,3	34,6	**-0,77**	-1,62	**-1,35**	-10,50	**-1,16**	-7,71
Palmas	81,1	87,7	73,1	72,6	70,3	70,1	56,1	68,8	57,8	26,7	36,5	**-1,30**	-2,42	**-1,92**	-11,77	**-1,72**	-8,81
Porto Velho	78,0	76,2	68,0	70,4	70,2	63,1	55,3	60,9	59,9	25,5	35,9	**-1,15**	-0,38	**-1,82**	-10,71	**-1,62**	-7,59
Rio Branco	74,3	76,0	66,7	69,9	71,5	64,1	53,2	67,0	57,3	27,1	36,8	**-0,81**	-0,64	**-1,40**	-9,70	**-1,21**	-6,80
Sudeste^a^	68,4	76,4	64,1	70,3	71,4	70,7	61,0	59,8	59,0	25,8	35,8	**-0,64**	-3,63	**-1,29**	-13,59	**-1,10**	-10,60
Belo Horizonte	71,7	83,4	68,4	71,6	72,5	71,8	67,2	62,3	61,7	28,7	39,2	**-0,75**	**-3,52**	**-1,40**	-13,42	**-1,19**	-10,27
Rio de Janeiro	65,0	75,1	62,8	69,0	70,0	68,1	52,9	55,4	54,0	20,5	29,8	**-0,80**	-3,99	**-1,45**	-14,02	**-1,27**	-11,25
São Paulo	65,5	73,2	62,2	70,8	72,6	71,9	64,5	62,2	63,8	22,6	33,4	-0,28	-2,66	-1,08	-15,03	-0,87	-11,79
Vitória	71,4	74,0	62,9	69,8	70,3	71,0	59,3	59,4	56,5	31,4	40,7	**-0,74**	-4,33	**-1,23**	-11,88	**-1,05**	-9,08
Centro-Oeste^a^	72,6	78,5	67,8	70,3	72,5	70,6	61,7	61,0	62,8	27,4	38,1	**-0,76**	-2,43	**-1,45**	-13,03	**-1,24**	-9,83
Campo Grande	68,9	79,3	70,5	71,4	71,2	68,0	61,0	62,0	63,4	26,9	37,7	**-0,67**	-1,29	**-1,38**	-12,25	**-1,17**	-9,01
Cuiabá	73,1	77,9	67,3	69,7	74,6	68,5	59,9	61,4	63,2	27,1	38,0	**-0,71**	-1,42	**-1,41**	-12,25	**-1,20**	-9,00
Distrito Federal	68,4	76,6	65,3	69,8	72,7	74,9	57,8	59,3	61,4	29,1	39,5	**-0,81**	-3,90	**-1,45**	-13,57	**-1,24**	-10,48
Goiânia	79,8	80,0	68,1	70,6	71,4	71,2	68,1	61,5	63,0	26,5	37,3	**-0,84**	-3,12	**-1,56**	-14,07	**-1,34**	-10,83
Sul^a^	68,4	78,8	63,8	70,9	71,1	69,6	60,4	60,5	63,3	24,0	34,8	**-0,56**	-1,87	**-1,33**	-13,68	**-1,12**	-10,43
Curitiba	71,3	79,1	67,3	71,2	71,7	71,3	66,0	61,9	67,8	24,3	35,9	**-0,62**	-1,48	**-1,47**	-14,53	**-1,25**	-11,03
Florianópolis	69,4	78,7	61,9	71,5	71,6	68,5	57,6	59,5	60,7	25,1	35,5	**-0,68**	-2,14	**-1,37**	-12,82	**-1,17**	-9,71
Porto Alegre	64,5	78,7	62,2	70,1	70,1	69,0	57,5	60,2	61,4	22,5	32,9	-0,38	-1,98	-1,15	-13,67	-0,94	-10,54
Total^b^	71,5	76,6	65,0	70,6	71,2	69,0	57,9	61,2	58,7	24,1	34,2	**-0,78**	-2,78	**-1,46**	-13,15	**-1,26**	-10,12

^a^ Valores médios das capitais de cada região.

^b^ Valores médios das 26 capitais e do Distrito Federal.

^c^ Dados apenas para o cadastro de telefonia fixa em 2023.

^d^ Dados apenas para o cadastro de telefonia móvel em 2023.

^e^ Dados combinados do cadastro de telefonia fixa e móvel em 2023.

Valores com significância estatística estão apresentados em negrito (p < 0,05).

Quanto aos cadastros fixos, maior intensidade de redução da taxa de resposta foi observada nas capitais da região Norte do País (-1,05 pp/ano; p < 0,001), apresentando tendência estável em todas as regiões no período mais recente (Tabela 2).

### Taxa de Recusa

No conjunto completo do período analisado, a evolução é favorável quanto à taxa de recusa, com valores variando de 9,1% em 2006 a 5,3% em 2023, quando considerado apenas o cadastro de telefones fixos. Valores semelhantes de recusa foram observados também entre os telefones móveis (4,1%). Em ambos os cenários, a redução média da taxa de recusa no período ficou em torno de 0,23 pp/ano (p = 0,030) (Tabela 3).

### Dias e Horários de Maior Realização de Contatos

De forma geral, no ano de 2023, as entrevistas realizadas pelo Vigitel se concentraram mais em dias de semana, quando comparados aos finais de semana, tanto para telefones fixos quanto para móveis (Figura 1). Considerando o horário das ligações, o maior número de contatos atendidos se deu no horário entre 9h e 20h para telefones fixos e móveis (Figura 2). Dados acerca do número de contatos atendidos também estão descritos segundo dia da semana (Figura 1) e horário (Figura 2).

**Figura 1:** Número de contatos atendidos nas capitais dos estados brasileiros e no Distrito Federal, por dia da semana para o cadastro de telefones fixos e móveis. Vigitel Brasil, 2023.

FDS: contatos aos finais de semana, sábado e domingo, e feriados.

Nota: A escala indica pelas cores mais frias (esverdeadas) os dias de semana com maior número de contatos atendidos, enquanto as cores mais quentes (avermelhadas) indicam aqueles com menor número contatos atendidos. Os dados não consideram a diferença na distribuição de entrevistadores entre os períodos do dia. Um total de 60 entrevistadores participou da operação, sendo 40 no período 8h45 às 15h15 e 20 no período das 15h15 às 21h30. Aos finais de semana, um turno único de entrevistadores trabalhava das 9h20 às 15h40.

**Figura 2:** Número de contatos atendidos nas capitais dos estados brasileiros e no Distrito Federal, por horário do dia para o cadastro de telefones fixos e móveis. Vigitel Brasil, 2023.

^1^ Contatos fora do horário de atendimento ocorrem apenas mediante solicitação expressa do entrevistado.

Nota: a escala indica pelas cores mais frias (esverdeadas) os horários do dia com maior número de contatos atendidos, enquanto as áreas mais quentes (avermelhadas) indicam aqueles com menor número contatos atendidos. Os dados não consideram a diferença na distribuição de entrevistadores entre os períodos do dia. Um total de 60 entrevistadores participou da operação, sendo 40 no período 8h45 às 15h15 e 20 no período das 15h15 às 21h30. Aos finais de semana, um turno único de entrevistadores trabalhava das 9h20 às 15h40.

## DISCUSSÃO

Este estudo analisou a tendência dos indicadores da operação do Vigitel em suas 17 edições, revelando um cenário desafiador. A taxa de elegibilidade diminuiu no período completo, e mais acentuadamente nos últimos anos, quando a redução foi quase quatro vezes a do período total. Apesar da inclusão de telefones móveis no cadastro em 2023 ter atenuado a queda da elegibilidade, a redução ainda se manteve elevada. A taxa de resposta também diminuiu no período completo e, conjuntamente com a queda da elegibilidade, representa um obstáculo para a sustentabilidade do inquérito. Em contrapartida, a taxa de recusa diminuiu no período completo, mostrando a consistente qualidade na operação do sistema. Nesse contexto, nosso estudo fornece uma análise atualizada e abrangente sobre questões metodológicas importantes para a melhoria da qualidade e continuidade do Vigitel.

As taxas de elegibilidade apresentaram grande redução desde o início do sistema, com valores entre 60% e 70% representando a moda entre 2007 e 2017, seguido de acentuado declínio. A ocorrência de linhas inelegíveis, especialmente devido à defasagem do cadastro, deve ser aceita como fenômeno natural, uma vez que o sorteio das linhas a serem chamadas durante toda a operação é feito em um único momento. Contudo, a intensa redução da taxa de elegibilidade, de 72,5% para 5,3% em 2023 para telefones fixos, expõe uma importante fragilidade dos inquéritos por telefone com quadro único de coleta de dados.

No Vigitel 2023, 61,3% das linhas telefônicas fixas e 54,5% no caso das linhas móveis não elegíveis corresponderam a linhas para as quais não foi possível contato após seis ou mais tentativas de contato em dias e horários distintos (dados não apresentados). Ressalta-se que a metodologia empregada para o cálculo das taxas do Vigitel (AAPOR #2) trata como inelegíveis aquelas linhas para as quais não foi possível identificar de forma definitiva a sua elegibilidade^16^. Este aumento no número de linhas inacessíveis somado à baixa cobertura pode reduzir a qualidade dos dados coletados, especialmente em regiões com cobertura reduzida, como Nordeste e Norte. Esses problemas também impactam no custo da pesquisa, aumentando o número de ligações e tentativas de triagem^18,19^.

Ressalta-se que mesmo com as grandes quedas observadas nos últimos anos, o Vigitel apresenta taxas de elegibilidade superiores àquelas do *Behavioral Risk Factor Surveillance System* (BRFSS), conduzido pelo *Centers for Disease Control and Prevention* (CDC), nos Estados Unidos^20^. Cabe considerar, no entanto, que o cálculo das taxas de elegibilidade aplicadas nesses inquéritos se difere um do outro. No Vigitel (AAPOR #2), indivíduos que responderam ou não a pesquisa compõem o denominador do cálculo. Já no BRFSS (AAPOR #4), temos no cálculo da elegibilidade apenas aqueles que não completaram a pesquisa, excluindo-se aqueles que responderam^16^. Tais diferenças metodológicas limitam a comparabilidade dos referidos inquéritos.

Alguns fatores estão relacionados a esses resultados, como o rápido aumento da utilização de celulares e da internet pela população^18^, sugerindo também que entrevistas telefônicas (por voz) devam coexistir com entrevistas na Internet (por dados)^21^. Assim, alguns inquéritos estão migrando para celulares, internet ou adotando modalidades mistas para compensar a falta de cobertura de linha fixa e a não resposta^19,22^. Destaca-se, entretanto, que cerca de 40% das linhas inelegíveis se encaixam em categorias de linha empresarial ou comercial, fora de serviço, inexistente e casa vazia de temporada ou veraneio, o que estaria mais relacionado a fragilidades da qualidade do cadastro do que a movimentos sociais alheios ao controle do inquérito.

É amplamente reconhecido que as taxas de resposta em inquéritos por telefone estão se reduzindo com o tempo, à medida que a “não resposta” aumenta^18^. No presente estudo, observou-se resultados inferiores para taxa de resposta de telefones móveis comparados aos fixos. O Vigitel 2023 foi a primeira edição a incorporar telefones móveis em seu cadastro. Desde 1984, o BRFSS é considerado o maior sistema de inquéritos telefônicos no mundo. O BRFSS adotou, a partir de 2011, o uso de entrevistas por telefone celular, complementando as entrevistas por telefone fixo, o que melhorou a validade, qualidade e representatividade da pesquisa. O cenário da pesquisa é ainda menos favorável do que o Vigitel, pois, em 2022, a taxa de resposta combinada (celular + fixo) foi de 45,1%^23^. Uma vez que o telefone móvel é dotado de mecanismo identificador de chamada e processos eletrônicos para bloqueio de chamadas indesejadas, apenas entrevistas concluídas no primeiro contato costumam ser bem-sucedidas, com uma proporção ínfima de agendamentos sendo convertidos em sucesso^19^.

Para 2023, o cenário identificado sugere redução de qualidade na operação, possivelmente em decorrência do pouco tempo disponível para implementação, pelo formato remoto da coleta, pela gradativa incorporação de novos entrevistadores, pela má qualidade do cadastro empregado e pelo período reduzido para a coleta de um volume significativo de entrevistas (a coleta de dados aconteceu em cerca de quatro meses – entre dezembro de 2022 e abril de 2023). Enquanto as operações até 2020 contavam com cerca de 40 entrevistadores, a de 2023 demandou cerca de 60.

Embora o contato telefônico tenha se tornado mais difícil, considerando as taxas de elegibilidade e de sucesso, a taxa de recusa do Vigitel se configura como o indicador que possivelmente melhor expressa a qualidade da operação do sistema, e tal indicador apresenta evolução favorável, reduzindo ao longo dos anos. Cabe destacar que em 13 dos 17 anos em estudo a taxa de recusa ficou abaixo de 5%, valor muito expressivo para inquéritos dessa natureza. Os valores apresentados para a taxa de recusa se referem tanto à recusa referente ao agendamento (obtida no contato inicial junto ao domicílio, a qual não possibilita nem que um morador da casa seja sorteado), quanto àquela referente à entrevista (quando um morador do domicílio já foi sorteado e esse se recusa a participar do estudo). Contrariamente aos resultados apresentados, estudos têm indicado aumento nas taxas de recusa em inquéritos telefônicos, o que está fortemente associado à triagem inicial do entrevistador^18,19^. Assim, a diminuição da taxa de recusa revela a prioridade do entrevistador em obter a cooperação do entrevistado, o que pode estar relacionado com a experiência adquirida ao longo de mais de uma década de entrevistas, especialmente através de formação e supervisão dos entrevistadores^13^.

A análise dos indicadores da operação Vigitel possibilita concluir sobre a fadiga da metodologia empregada, com redução na elegibilidade e na taxa de resposta. Tal contexto é comum na realização de inquéritos populacionais que precisam se adequar periodicamente às mudanças sociais e tecnológicas^21^. Salva-se, no presente momento, apenas a taxa de recusa que, a despeito de estar longe de seus menores níveis, permanece em patamares satisfatórios. É perceptível que a participação de inquéritos telefônicos como único método de coleta de dados tem se tornado menos conveniente na medida em que trocas de informação por dados (como serviço de mensagens curtas – SMS e plataformas de mensagem automática) se tornam mais frequentes^21^. A busca de alternativas que façam a participação mais conveniente para o respondente é, sem dúvida, um caminho constante para inquéritos dessa natureza.

Com base nestes resultados, não é possível inferir sobre a magnitude de influência de cada um desses fatores na qualidade geral do inquérito, ficando essa conclusão condicionada à ocorrência de uma nova operação do Vigitel nas condições ideais de planejamento e execução, como aconteceram em anos anteriores^13^. Ainda, acredita-se que o inquérito se beneficia com a inclusão de novo modal para coleta, dessa forma, a continuidade de estudos que permitem acompanhar o atual dinamismo tecnológico será importante para a manutenção do inquérito.

É importante destacar a consolidação do Vigitel enquanto sistema de vigilância dos fatores de risco e proteção para as DCNT no Brasil, se configurando como exemplo de iniciativa de coleta de dados com boa qualidade, custo reduzido e grande agilidade, pioneira em países de renda média e baixa. Ressalta-se que a sustentabilidade na estrutura de coleta de dados é uma etapa fundamental para a retomada do sistema em direção adequada, bem como a continuidade de estudos capazes de apontar e incorporar melhoras à sua metodologia.

**Tabela 3:** . Percentual (%) de recusa* dentre as linhas telefônicas elegíveis sorteadas nas capitais dos estados brasileiros e no Distrito Federal, por ano. Vigitel Brasil, 2006 a 2023.

**Localidade**	**Ano**											**Variação média no período (pp)**					
	**2006**	**2009**	**2012**	**2015**	**2018**	**2019**	**2020**	**2021**	**2023^c^**	**2023^d^**	**2023^e^**	**2006–2023^c^**	**2018–2023^c^**	**2006–2023^d^**	**2018–2023^d^**	**2006–2023^e^**	**2018–2023^e^**
Nordeste^a^	8,8	3,1	9,1	2,3	4,1	1,7	0,4	2,4	5,3	4,1	4,4	**-0,22**	1,27	**-0,25**	0,90	**-0,24**	0,99
Aracaju	11,3	4,1	9,1	2,9	4,2	1,2	0,1	2,0	6,5	5,0	5,4	**-0,29**	1,78	**-0,32**	1,33	**-0,31**	1,45
Fortaleza	10,1	3,6	8,7	2,1	3,7	1,3	0,6	2,6	4,9	3,7	4,1	**-0,30**	1,25	**-0,32**	0,91	**-0,32**	1,01
João Pessoa	10,3	3,3	11,9	3,2	4,7	2,5	0,5	2,2	6,1	3,8	4,4	-0,24	1,24	**-0,28**	0,55	-0,27	0,74
Maceió	7,2	3,0	9,9	2,0	4,6	1,7	0,5	2,6	3,8	4,6	4,3	-0,22	0,84	-0,21	1,07	-0,21	0,99
Natal	7,8	2,5	8,3	1,8	4,4	1,8	0,5	2,2	5,1	3,6	4,1	-0,17	1,15	-0,20	0,71	-0,19	0,84
Recife	8,7	2,9	9,8	2,4	4,1	1,8	0,2	2,6	3,6	5,0	4,6	**-0,29**	0,78	-0,26	1,23	**-0,27**	1,10
Salvador	7,6	2,2	5,8	2,1	3,0	1,4	0,3	2,3	4,6	3,6	3,9	**-0,19**	1,16	**-0,21**	0,85	**-0,20**	0,95
São Luís	6,9	2,8	8,4	2,6	4,9	1,9	0,4	2,4	6,3	4,6	5,0	-0,11	1,51	-0,14	0,99	-0,13	1,14
Teresina	9,1	3,1	9,5	1,8	3,7	1,9	0,3	2,4	6,8	2,6	3,6	-0,21	1,68	**-0,29**	0,43	**-0,27**	0,72
Norte^a^	7,2	1,9	8,3	2,1	4,6	1,0	0,2	1,7	5,3	3,3	3,8	-0,12	1,43	-0,16	0,83	-0,15	1,00
Belém	8,5	3,2	9,3	1,8	7,5	0,7	0,3	1,9	6,8	3,2	4,1	-0,18	1,97	-0,25	0,91	-0,24	1,17
Boa Vista	7,2	1,5	8,1	2,6	4,5	1,5	0,2	1,5	4,2	3,0	3,4	-0,14	0,94	-0,16	0,60	-0,15	0,69
Macapá	7,2	2,3	10,4	2,8	5,7	0,7	0,2	1,4	4,2	2,4	2,9	-0,16	1,15	-0,19	0,62	-0,18	0,77
Manaus	7,9	1,5	12,7	2,3	4,3	0,8	0,2	1,1	5,9	2,4	3,5	-0,16	1,64	-0,23	0,58	-0,21	0,91
Palmas	5,4	0,2	4,2	1,5	3,8	1,0	0,2	2,1	4,5	4,5	4,5	-0,03	1,23	-0,03	1,23	-0,03	1,23
Porto Velho	7,5	2,2	6,0	1,8	3,0	1,1	0,3	2,4	6,5	3,6	4,4	-0,07	1,81	-0,12	0,94	-0,11	1,20
Rio Branco	6,8	2,1	7,3	1,9	3,1	1,1	0,3	1,6	4,9	3,8	4,1	-0,09	1,26	-0,12	0,93	-0,11	1,03
Sudeste^a^	10,8	4,2	8,0	3,7	4,6	2,9	0,6	2,9	4,4	5,3	5,0	**-0,35**	0,68	**-0,33**	0,95	**-0,34**	0,87
Belo Horizonte	9,8	3,7	4,9	2,3	3,9	2,3	0,3	2,9	4,0	4,9	4,6	**-0,27**	0,77	**-0,25**	1,06	**-0,26**	0,97
Rio de Janeiro	13,3	5,6	10,6	6,5	5,5	4,3	0,6	2,9	4,3	5,5	5,2	**-0,43**	0,23	**-0,41**	0,59	**-0,41**	0,49
São Paulo	11,3	3,6	8,2	2,2	4,2	2,2	0,6	3,0	4,2	4,7	4,6	**-0,43**	0,83	**-0,42**	1,01	**-0,42**	0,96
Vitória	9,0	3,6	8,0	3,8	4,7	2,9	0,8	2,9	5,2	6,1	5,7	**-0,26**	0,88	**-0,25**	1,15	**-0,25**	1,05
Centro-Oeste^a^	9,3	3,6	7,2	3,6	3,4	2,1	0,4	2,6	6,5	4,5	5,1	-0,14	1,53	-0,18	0,93	-0,17	1,11
Campo Grande	7,0	0,9	8,3	1,7	3,2	2,6	0,3	2,7	7,9	2,9	4,4	-0,05	1,83	-0,15	0,31	-0,12	0,76
Cuiabá	7,7	2,4	6,8	3,2	3,2	2,3	0,2	2,5	9,2	5,6	6,7	0,00	2,30	-0,07	1,23	-0,05	1,55
Distrito Federal	13,8	7,2	7,5	5,3	3,5	1,9	0,6	2,5	4,9	4,7	4,8	-0,30	1,10	-0,30	1,03	-0,30	1,06
Goiânia	9,0	3,9	6,1	4,3	3,5	1,7	0,5	2,6	3,9	4,8	4,5	**-0,22**	0,89	**-0,20**	1,14	**-0,21**	1,07
Sul^a^	12,2	3,1	11,5	4,0	5,9	3,1	0,7	2,8	4,8	4,7	4,7	-0,28	0,71	-0,28	0,69	-0,28	0,67
Curitiba	8,9	2,8	10,0	2,9	4,4	2,3	0,4	2,8	5,0	4,8	4,9	-0,18	1,04	-0,18	1,00	-0,18	1,01
Florianópolis	12,7	4,3	10,9	5,4	6,8	3,4	0,9	2,8	5,3	5,0	5,1	**-0,32**	0,77	**-0,33**	0,65	**-0,32**	0,69
Porto Alegre	15,0	2,0	13,5	3,7	6,7	3,6	0,9	2,9	4,0	4,3	4,2	-0,35	0,32	-0,34	0,40	-0,34	0,38
Total^b^	9,1	3,0	8,7	2,8	4,4	1,9	0,4	2,4	5,3	4,1	4,5	-0,21	1,19	**-0,23**	0,86	**-0,23**	0,95

^a^ Valores médios das capitais de cada região.

^b^ Valores médios das 26 capitais e do Distrito Federal.

^c^ Dados apenas para o cadastro de telefonia fixa em 2023.

^d^ Dados apenas para o cadastro de telefonia móvel em 2023.

^e^ Dados combinados do cadastro de telefonia fixa e móvel em 2023.

*Recusa global, envolvendo tanto recusas relacionadas à entrevista quanto aquelas relacionadas ao agendamento (maiores informações na seção Métodos).

Valores com significância estatística estão apresentados em negrito (p < 0,05).
